# Duodenojejunostomy following failed gastrojejunostomy in superior mesenteric artery syndrome: A case report

**DOI:** 10.1016/j.ijscr.2024.109380

**Published:** 2024-02-10

**Authors:** Parsa Yazdanpanahi, Alireza Keshtkar, Farnaz Atighi, Mehdi Foroughi

**Affiliations:** a Student Research Committee, School of Medicine, Shiraz University of Medical Sciences, Shiraz, Iran; b Department of Pediatric Surgery, Namazee Hospital, Shiraz University of Medical Sciences, Shiraz, Iran

**Keywords:** Superior mesenteric artery syndrome (SMA syndrome), Gastrojejunostomy, Duodenojejunostomy, Case report

## Abstract

**Introduction:**

Superior mesenteric artery (SMA) syndrome is a rare duodenal-vascular anatomic disorder leading to external compression on the duodenum. The first step of treatment usually is conservative, and in the case of failure, surgical management is the treatment choice. Treatment success with duodenojejunostomy after failure in gastrojejunostomy can show the uniqueness of this article.

**Case presentation:**

A 14-year-old boy came to our hospital with a complaint of epigastric pain, nausea, bilious vomiting, and weight loss since 6 months ago. Conservation therapy and laparotomic Braun anastomosis and gastrojejunostomy was performed due to the SMA syndrome diagnosis 2.5 months before the admission. At our hospital, an alteration of gastrojejunostomy by duodenojejunostomy employing a diamond-shaped anastomosis between the third portion of the duodenum (D3) and a part of jejunum that was placed 15 cm away from the ligament Treitz was done. A significantly dilated stomach and the first three parts of the duodenum were observed during the procedure. After the second surgical intervention, the general condition of the patient dramatically improved.

**Clinical discussion:**

Conservative treatment, including nasogastric tube decompression, postural changes, and nutritional support with hyperalimentation, has a variable success rate. However, in some cases, surgery may be necessary. Surgeons prefer laparoscopic duodenojejunostomy due to its outstanding success rate, ranging from 80 % to 100 %. But, in some case reports it is suggested that gastrojejunostomy could be done in cases with severe duodenal dilation instead of duodenojejunostomy. The initial gastrojejunostomy failed because of ongoing symptoms, which was finally revised with a duodenojejunostomy.

**Conclusion:**

It is suggested to use duodenojejunostomy after failure of gastrojejunostomy or it can be employed as the first surgical option even in cases with severe dilation. Because it is a more efficient correction with fewer complications than gastrojejunostomy.

## Highlights

• Duodenojejunostomy following a failed gastrojejunostomy in superior mesenteric artery (SMA) syndrome.
• Duodenojejunostomy effectively treats SMA syndrome after gastrojejunostomy failure.
• Duodenojejunostomy as a first line treatment option for SMA syndrome.
• Duodenojejunostomy can be mentioned as a more cost-effective intervention.
• Duodenojejunostomy can be used if there is no improvement.

## 1 Introduction

Superior Mesenteric Artery (SMA) syndrome, also known as Wilkie's syndrome, chronic duodenal ileus, Cast syndrome, Arteriomesenteric duodenal obstruction, chronic duodenal obstruction, is a rare duodeno-vascular disorder first described by Carl van Rokitansky in the 1840s. He introduced it as a phenomenon that causes proximal intestinal dilation by creating compression on the third part of the duodenum which is as a result of a tight angle between the abdominal aorta and the superior mesenteric artery [1,2]. The superior mesenteric artery (SMA) and aorta normally form an angle of about 25–60 degrees. Adults with an aortomesenteric angle of less than 25 degrees are thought to have superior mesenteric artery (SMA) syndrome [3]. The prevalence of this syndrome ranges from 0.013 to 0.3, with a higher incidence in females. [4,5]. The loss of retroperitoneal fat can lead to the third part of duodenum obstruction due to external pressure from the superior mesenteric artery (SMA), resulting in dilation of the first and second parts of the duodenum and the stomach. Significant weight loss, eating disorders, congenital anatomical variance, prolonged cast of the body, trauma, burns, cancer, and substance misuse are other causes of this syndrome [1].

The first step for the treatment of these patients is conservative management, and in the case of failure, surgical management is used in the next step, for which various methods have been introduced, including the release of the ligament of Treitz (Strong's Procedure), Lad's procedure, anterior transposition of the duodenum, duodenojejunostomy, and gastrojejunostomy [6]. Strong's procedure can reduce the obstruction without anastomosis by mobilization of the duodenum [7]. In duodenojejunostomy, the adhesive bands are divided, and the jejunum is connected to the duodenum in such a way that the obstructed part is bypassed [8]. Gastrojejunostomy is a type of gastric/intestinal bypass procedure that directly connects the gastric pouch to the jejunum. The long-term side effects of this operation can be mentioned as dumping syndrome, anastomosis stenosis, blind loop syndrome, marginal ulcer, and bile and pancreatic enzymes reflux. Although duodenojejunostomy is technically more difficult than gastrojejunostomy, this procedure is more physiological and does not involve the chance of bile and pancreatic enzymes reflux and its complications which are frequently observed in gastrojejunostomy [6].

In this case report, we presented a patient who underwent a duodenojejunostomy after failure to improve from gastrojejunostomy. There are few articles that report two types of operations performed on the same patient with SMA syndrome. The success of treatment with duodenojejunostomy after failure in gastrojejunostomy can show the uniqueness of this article.

## 2 Case presentation

**Fig. 1:**
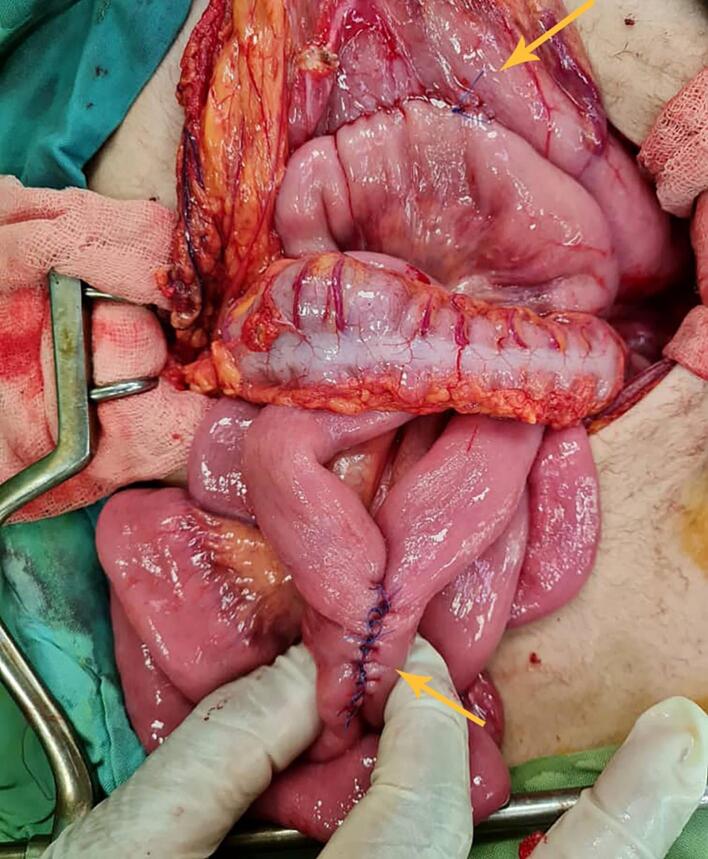
The site of gastrojejunostomy was shown by upper arrow and the Braun anastomosis is marked by lower arrow.

**Fig. 2:**
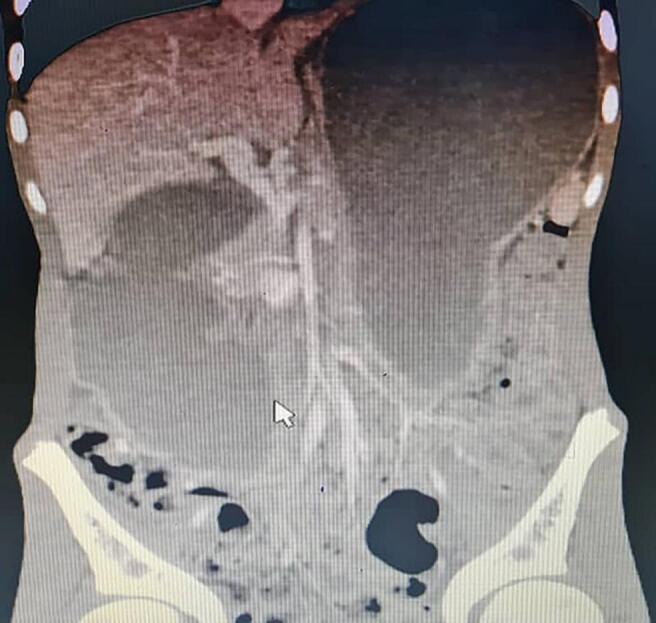
The initial CT scan shows the obstructed view.

**Fig. 3:**
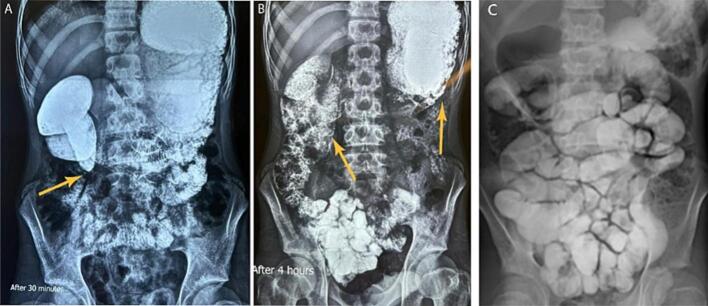
In the 30-min and 4-h images, the stomach and D1, D2, and D3 of the duodenum were shown very dilated. C: the post operative Gi series of the patient after two hours.

**Fig. 4:**
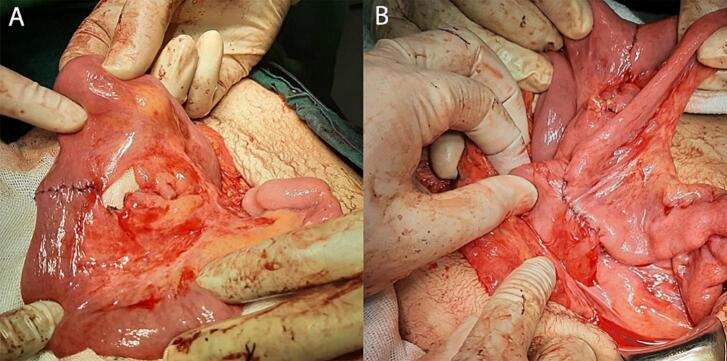
The end-to-end jejunojejunostomy was applied with prolene 4–0 and reinforced with lambert 5–0. The site of anastomosis was 15 cm away from the Treitz ligament (A). The first part of D3 was opened horizontally and was anastomosed to the loop of jejunum which had been cut longitudinally.

A 14-year-old boy was referred to our hospital with complaints of epigastric pain, nausea, bilious vomiting, asthenic habitus, and weight loss. The patient was diagnosed with SMA syndrome (Fig. 2) about six months prior to this admission and due to the lack of condition improvement after conservative therapy for 4 months and a weight loss of about 15 kg (45 to 30 kg), gastrojejunostomy and Braun anastomosis jejunojejunostomy were performed for him in another hospital. Unfortunately, the patient's symptoms continued after this surgery for 2.5 months which made the patient come to our hospital (Fig. 1). At this admission, abdominal tenderness was not present in the physical examination and epigastric pain was aggravated in the supine position. In the laboratory tests, complete blood count, electrolytes, and the renal function tests and liver function were within the normal range. Urine culture was positive with enterococci and antibiotic therapy was started for him. In the radiological imaging of the upper gastrointestinal (GI) series stomach, D1, D2, and D3 were severely dilated, and no sign of complete obstruction was detected (Fig. 3). In the upper gastrointestinal endoscopy, the gastrojejunostomy anastomosis was patent and the body of the stomach was full of bilious fluid. Due to persistent nausea, vomiting, and severe weight loss, the decision was made for surgical intervention. The patient went to the operating room, gastrojejunostomy was taken down by stapler and 2 two NO 55 cartridge and site of stomach suture was reinforced with prolene 3–0 in two layers, first layer was continues suture and second layer was Lambert suture with prolene 4–0, Braun anastomosis was resected, and duodenojejunostomy between D3 and jejunum was applied with a single layer side-to-side diamond-shaped anastomosis using Gambee suture with PDS and prolene 4–0 and reinforced by Lambert suture with prolene 5–0 (Fig. 4). After the second surgery, the general condition of the patient was improved, nausea and vomiting were resolved, and the patient's weight increased by about 10 kg. In the upper GI series that was performed 3 months after the operation, D2 was slightly dilated, the C-loop was normal, and the jejunum was slightly dilated and located on the right side of the abdomen (Fig. 3). The contrast material entered the colon after 2 h. the work has been reported in line with the SCARE criteria [9] Fig. 3, Fig. 4.

## 3 Discussion

The SMA syndrome is diagnosed by the high clinical suspicion and radiological findings of duodenal obstruction in imaging investigations. Imaging techniques that are employed to confirm the diagnosis are contrast radiography, computed tomography (CT), magnetic resonance imaging (MRI), abdominal sonography (US), endoscopy, and endoscopic sonography (EUS) [1,5].

Previous studies have emphasized the need to individualize the management of SMA syndrome according to patients' characteristics, either by conservative approaches or surgical interventions [5,10]. Conservative measures including nasogastric tube decompression, postural changes, fluid resuscitation, correcting electrolyte abnormalities, and prokinetic medications aim to resolve the symptoms of duodenal obstruction. Then providing high-calorie nutritional support through different routes, such as oral, parenteral, or enteral feeding can help patients regain weight and increase the adipose tissue around the duodenum, which can relieve the external compression from the decreased angle between the abdominal aorta and the superior mesenteric artery [1].

Investigations have found a success rate between 14 % and 83 % for conservative management of SMA syndrome [11,12]. Moreover, a study has revealed that in about 70 % of patients, it would be necessary to opt for earlier surgical intervention to bypass the obstructed parts [13]. However, recent works have publicized a lower requirement for operation (11.5 %), most probably attributable to the advances in nutritional administrations [14]. Therefore, there is a lack of consensus evidence for the optimal interval or indication for conservative therapy. In our case, despite four months of non-operative treatment, his condition didn't improve, and he experienced further weight loss; therefore, he was a candidate for surgical intervention. It is recommended that surgical treatment be indicated only after failed initial management, especially in the presence of a dramatic anatomic changes, like dilatation of the stomach and duodenum.

Several surgical modalities, utilizing either a laparotomic, laparoscopic or a robotic method, are available for management of SMA syndrome. The most prevalent anastomotic surgical interventions are gastrojejunostomy, gastroduodenostomy, and duodenojejunostomy. Moreover, the dissolution of the Treitz ligament (Strong's technique) or retroperitoneal duodenal connections (Ladd's technique) offer the opportunity of duodenal relocation and reorientation that alleviate duodenal constriction through anastomosis-free modalities [1,6]. Lately, a successful EUS-guided gastroenterostomy has been executed for the treatment of the SMA syndrome [15].

Laparoscopic duodenojejunostomy is currently the most common choice among surgeons due to its high success rate (80 %–100 %), short convalescence course after surgery, and minimal complications [10]. Also, duodenojejunostomy causes fewer changes in the gastrointestinal normal anatomy and can preserve a physiologic construct. Gastrojejunostomy is no longer a commonly used surgical technique since incomplete proximal gastrointestinal obstruction relief and continuation of symptoms after this surgery have been reported. Complications such as the development of blind loop syndrome, bile reflux gastritis, peptic ulceration, and gastric malignancy caused by significant bilious reflux, and anastomotic hemorrhage that may necessitate additional surgical correction mostly with duodenojejunostomy in certain cases [4,5].

The appropriate surgical strategy can be adopted by surgeons during laparotomic surgeries according to the laparotomic exploration findings, anatomical variations, and safety considerations. In our case, loop gastrojejunostomy accompanied by Braun anastomosis, a less conventional surgical option in developed countries, was performed for him at first due a severe dilated and not suitable for surgical anastomosis duodenum. Gastrojejunostomy is a common technique that is performed in developing countries like Iran due to its unchallenging and fast technique. Brown anastomosis is designed to reduce reflux from the ascending limb by changing the path of pancreatic and bile enzymes. Since the pyloric valve in these patients usually functions normally, it can prevent reflux. Over time, as the patient's physical condition and weight improve, the degree of stenosis decreases [16]. Although gastrojejunostomy can bypass duodenal obstruction and Braun enteroentrostomy is created to alleviate the bilious reflux into the stomach, this patient was referred to our hospital due to the continuation of nausea, vomiting, and bile reflux during a 2.5-month follow-up of post-gastrojejunostomy as a result of incomplete bile diversion from the stomach through Braun jejunojejunstomy. In the operating room, he had a very grossly dilated stomach, which led to gastroparesis and delayed emptying of the stomach which made the gastric dilatation worsen. Stomach distention has been reported in several SMA syndrome cases but before surgical intervention [[17], [18], [19]].

The considerable point in this case is that due to the severe dilatation of the second part of the duodenum (D2), a duodenojejunostomy was created in a new method with a diamond-shaped anastomosis between the third part of the duodenum (D3) and the jejunum. The diamond-shaped anastomosis which is commonly performed in congenital duodenal obstruction patients is made by a transverse incision in the dilated proximal duodenum, and a longitudinal incision in the duodenum distal to the obstruction [20]. In our case, the first part of D3 was opened horizontally, a loop of jejunum was opened longitudinally, and anastomosis between these parts was completed. In conclusion, we report a failed duodenal obstruction relief after gastrojejunostomy with Braun jejunojejunostomy. Therefore, additional surgical revision of gastrojejunostomy and duodenojejunostomy was necessitated in this case.

By reviewing this case, it is suggested that in developing countries such as Iran, where gastrojejunostomy is commonly chosen for the surgical intervention of SMA syndrome, duodenojejunostomy can be used if there is no improvement, or duodenojejunostomy can be employed as the first surgical option. Gastrojejunostomy can be preferably employed to eradicate the upper digestive system obstruction when the duodenum is unsuitable for anastomosis because of inflammation, excessive dilatation of the duodenum, local malignant tumors and ulcers, and presence of dense adhesional band [21,22]. Also, duodenojejunostomy can be mentioned as a more cost-effective intervention because it reduces the need for re-operation and hospitalization even in complicated cases.

## 4 Conclusion

Duodenojejunostomy can be utilized as the optimal surgical modality owing to its efficacy and reduced complications compared to gastrojejunostomy, although when duodenum is unfit for anastomosis gastrojejunostomy is the preferred method.

## Abbreviations

SMA syndrome: superior mesenteric artery syndrome
D1: first part of duodenum
D2: second part of duodenum
D3: third part of duodenum
GI series: gastrointestinal series
kg: kilogram

## Consent for publication

Written informed consent was obtained from the patient to publish this Case series. A copy of the written consent is available for review and can be requested at any time by the journal's editor.

## Ethical approval

Our study has been reviewed and approved by the Medical Ethics Committee of Shiraz University of Medical Sciences. The study is exempt from ethnical approval in our instutation.

## Funding

None.

## Author contribution

MF designed the study and revised the manuscript. PY, AK, and FA were in charge of collecting data and writing the manuscript. All authors read and approved the final manuscript.

## Guarantor

Corresponding author.

Information of the patients can be asked from the authors. Don't hesitate to get in touch with the corresponding author if you are attracted to such data.

## Conflict of interest statement

The authors declare that they have no competing interests.

## Data Availability

Information of the patients can be asked from the authors. Don't hesitate to get in touch with the corresponding author if you are attracted to such data.

## References

[1] Oka A., Awoniyi M., Hasegawa N., Yoshida Y., Tobita H., Ishimura N., Ishihara S. (2023). Superior mesenteric artery syndrome: diagnosis and management. World J. Clin. Cases.

[2] Yale S.H., Tekiner H., Yale E.S. (2020). Historical terminology and superior mesenteric artery syndrome. Int. J. Surg. Case Rep..

[3] Konen E., Amitai M., Apter S., Garniek A., Gayer G., Nass S., Itzchak Y. (1998). CT angiography of superior mesenteric artery syndrome. AJR Am. J. Roentgenol..

[4] Lee C.S., Mangla J.C. (1978). Superior mesenteric artery compression syndrome. Am. J. Gastroenterol..

[5] Merrett N.D., Wilson R.B., Cosman P., Biankin A.V. (2009). Superior mesenteric artery syndrome: diagnosis and treatment strategies. J. Gastrointest. Surg..

[6] Mandarry M., Zhao L., Zhang C., Wei Z. (2010). Übersicht zum Arteria mesenterica superior-Syndrom. Eur. Surg..

[7] Konstantinidis H., Charisis C., Kottos P. (2018). Robotic Strong's procedure for the treatment of superior mesenteric artery syndrome. Int J Med Robot..

[8] Stavely A. (1908). Acute and chronic gastromesenteric ileus with cure in a chronic case by duodenojejunostomy. Bull. Johns Hopkins Hosp..

[9] Sohrabi C., Mathew G., Maria N., Kerwan A., Franchi T., Agha R.A., Collaborators. (2023). The SCARE 2023 guideline: updating consensus surgical CAse REport (SCARE) guidelines. Int. J. Surg..

[10] Lee T.H., Lee J.S., Jo Y., Park K.S., Cheon J.H., Kim Y.S. (2012). Superior mesenteric artery syndrome: where do we stand today?. J. Gastrointest. Surg..

[11] Ha C.D., Alvear D.T., Leber D.C. (2008). Duodenal derotation as an effective treatment of superior mesenteric artery syndrome: a thirty-three year experience. Am. Surg..

[12] Biank V., Werlin S. (2006). Superior mesenteric artery syndrome in children: a 20-year experience. J. Pediatr. Gastroenterol. Nutr..

[13] Burrington J.D. (1976). Vascular compression of the duodenum. Surgery.

[14] Wan S., Zhang L., Yang J., Gao X., Wang X. (2020). Superior mesenteric artery syndrome improved by enteral nutritional therapy: a retrospective case-series study in a single institution. Ann. Nutr. Metab..

[15] Storm A.C., Mahmoud T., Akiki K., Law R.J. (2022). Endoscopic ultrasound-guided Gastrojejunostomy for superior mesenteric artery syndrome secondary to rapid weight loss. ACG Case Rep. J..

[16] Xu B., Zhu Y.H., Qian M.P., Shen R.R., Zheng W.Y., Zhang Y.W. (2015). Braun enteroenterostomy following pancreaticoduodenectomy: a systematic review and meta-analysis. Medicine (Baltimore).

[17] Sakurai Y., Hirai F., Abe M., Okaya T., Suzuki H., Sugano I. (2020). A case of gastric ischemia caused by massive gastric dilatation due to superior mesenteric artery syndrome. Clin. J. Gastroenterol..

[18] Huang Z., Li C., Tang G. (2022). Superior mesenteric artery syndrome with acute gastric dilatation caused by binge eating in an adolescent. Korean J. Intern. Med..

[19] Loi C.M., Chen K.H. (2023). Total gastric necrosis following massive gastric dilatation due to superior mesenteric artery syndrome. Asian J. Surg..

[20] Kimura K., Tsugawa C., Ogawa K., Matsumoto Y., Yamamoto T., Asada S. (1977). Diamond-shaped anastomosis for congenital duodenal obstruction. Arch. Surg..

[21] Deshpande S.H., Thomas J., Chiranjeev R., Pandya J.S. (2021). Superior mesenteric artery syndrome in a patient with celiacomesenteric trunk. BMJ Case Rep..

[22] Kirby G.C., Faulconer E.R., Robinson S.J., Perry A., Downing R. (2017). Superior mesenteric artery syndrome: a single Centre experience of laparoscopic duodenojejunostomy as the operation of choice. Ann. R. Coll. Surg. Engl..

